# Time and Space in Tzeltal: Is the Future Uphill?

**DOI:** 10.3389/fpsyg.2012.00212

**Published:** 2012-07-09

**Authors:** Penelope Brown

**Affiliations:** ^1^Language Acquisition Department, Max Planck Institute for PsycholinguisticsNijmegen, Netherlands

**Keywords:** time, space, language and cognition, absolute frame of reference, metaphor, Tzeltal, Mayan

## Abstract

Linguistic expressions of time often draw on spatial language, which raises the question of whether cultural specificity in spatial language and cognition is reflected in thinking about time. In the Mayan language Tzeltal, spatial language relies heavily on an absolute frame of reference utilizing the overall slope of the land, distinguishing an “uphill/downhill” axis oriented from south to north, and an orthogonal “crossways” axis (sunrise-set) on the basis of which objects at all scales are located. Does this absolute system for calculating spatial relations carry over into construals of temporal relations? This question was explored in a study where Tzeltal consultants produced temporal expressions and performed two different non-linguistic temporal ordering tasks. The results show that at least five distinct schemata for conceptualizing time underlie Tzeltal linguistic expressions: (i) deictic ego-centered time, (ii) time as an ordered sequence (e.g., “first”/“later”), (iii) cyclic time (times of the day, seasons), (iv) time as spatial extension or location (e.g., “entering/exiting July”), and (v) a time vector extending uphillwards into the future. The non-linguistic task results showed that the “time moves uphillwards” metaphor, based on the absolute frame of reference prevalent in Tzeltal spatial language and thinking and important as well in the linguistic expressions for time, is not strongly reflected in responses on these tasks. It is argued that systematic and consistent use of spatial language in an absolute frame of reference does not necessarily transfer to consistent absolute time conceptualization in non-linguistic tasks; time appears to be more open to alternative construals.

## Introduction

In languages all over the world, when referring to abstract concepts of time speakers often utilize more concrete perceptual experience based metaphors of space. Some aspects of the experience of time are probably universal, for example time experienced as continuous unidirectional change marked by the appearance/disappearance of objects and the beginning/fulfillment of events (Boroditsky, 2001), giving rise to the widespread conceptualization of time as a one-dimensional vector on which time points can be expressed by spatial metaphors like “ahead” and “behind.” Another plausibly universal basis for construing the vector of time derives from the canonical way humans walk, facing forward, into later-occurring events (Clark, 1973; Traugott, 1975, 1978; Alverson, 1994; Haspelmath, 1997), and the cyclic recurrence of events (the sun rising, the seasons passing) is also universally apparent.

But certain aspects of time are underspecified by experience, leaving open the possibility of different construals. This applies in particular to the directional axis in which time as spatially construed moves: is it from back to front, down to up, left to right, east to west – or the reversal – or none of these? A number of scholars have pointed out crosslinguistic differences in time expressions and found evidence for corresponding differences in speakers’ conceptualizations of time (e.g., Whorf, 1954; Scott, 1989; Boroditsky, 2000, 2001; Núñez and Sweetser, 2006; Boroditsky et al., 2008, 2011; Casasanto and Boroditsky, 2008; Bender et al., 2010; Boroditsky and Gaby, 2010; Lai and Boroditsky, under review). Some have demonstrated that different linguistic metaphors for time can have a deep effect – even when not speaking – on cognitive construals of time (e.g., Casasanto et al., 2004; Casasanto, 2008).

**Figure 1:**
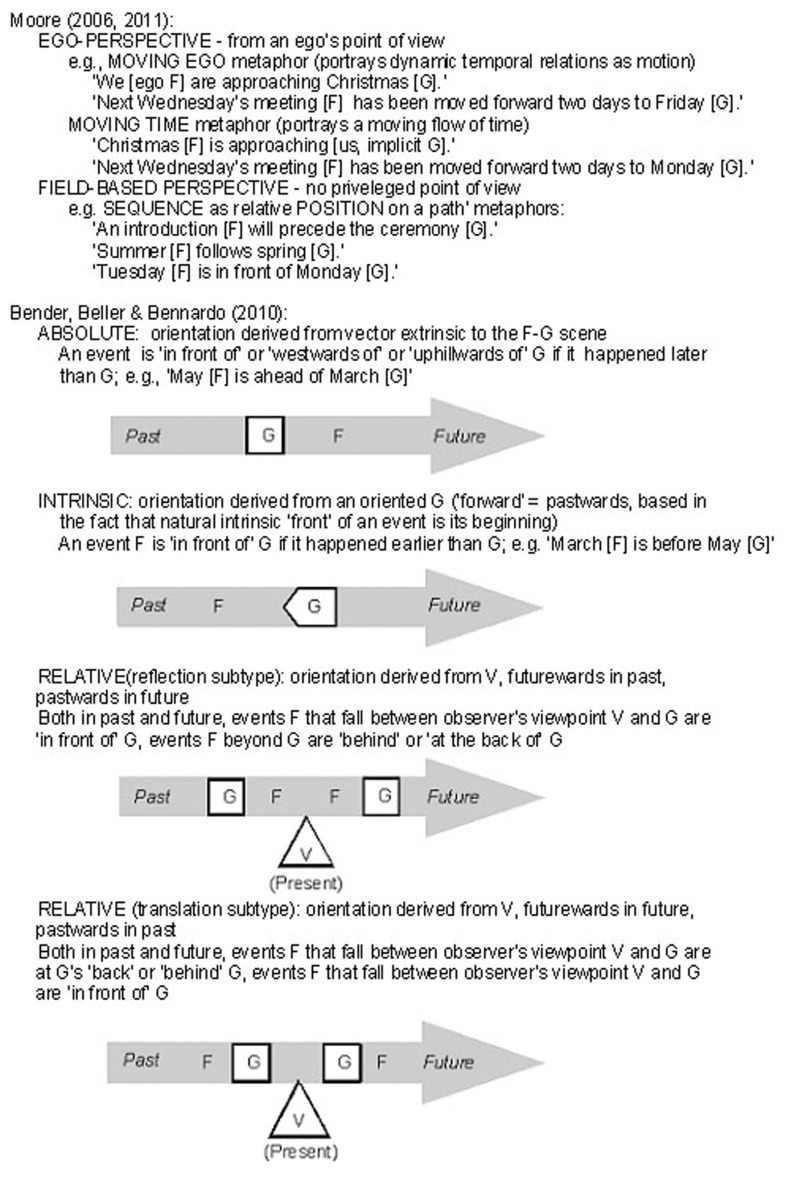
**Temporal frames of reference**.

Assessing different linguistic constructions of time requires a typology of the various within-language and crosslinguistically documented kinds of temporal framing. There is wide variation in the literature in the distinctions considered to be essential for characterizing frames of reference used in time reference and, as in the spatial frame of reference literature, considerable disagreement about how to capture the role of deictic anchoring. Adopting Talmy’s (2000) terminology of a figure-ground structure, where the figure (F) refers to the thing (person, object, or event) whose spatial or temporal location is being assessed relative to some reference point, the ground (G), we may distinguish two recent proposals. Moore (2006, 2011) makes a two-way distinction between ego-perspective (viewpoint dependent) and field-based perspective (viewpoint independent). Bender et al. (2010) make a four-way distinction based on an expansion of Levinson’s (2003) spatial frames of reference: absolute (vector extrinsic to the F–G configuration, viewpoint independent), intrinsic (object based vector, viewpoint independent), and relative (reflection subtype), a viewpoint based perspective where directional vectors are reflected symmetrically with past and future vectors *toward* deictic origo vs. relative (translation subtype), a viewpoint based perspective with past and future vectors *away from* deictic origo. A third proposal, the most elaborate to date, is that of Tenbrink (2011). She distinguishes 19 different spatial reference frames varying in the three dimensions of external/internal relationships between entities, static/dynamic, and absolute/intrinsic/relative; in the temporal domain these reduce to eight. Major distinctions captured in these proposals are exemplified in Figure 1.

The present study has two major aims: (1) describe the linguistic expressions for time in the Mayan language Tzeltal and characterize the frames of reference they utilize, and (2) test the hypothesis that the dominant patterns in spatial reference usage transfer to temporal frames of reference in this speech community.

### Space and time in tzeltal

This study addresses one type of crosslinguistic difference underlying expressions for time – differences in the preferred frame of reference for calculating vectors in terms of which spatial relations are assessed – and asks the question: Are such differences reflected in correspondingly different metaphors for, and construals of, time? Speakers of the Mayan language Tzeltal, as spoken in the rural community of Tenejapa in southeastern Mexico, habitually use an absolute (or “geocentric”) frame of reference based in the overall “downhill/uphill” slope of the land for describing locations and movements in both small-scale and distant space (Levinson, 2003). They also utilize an intrinsic (body part based) frame of reference, but no relative (projective left/right) frame of reference is in systematic use in this community. That is, there is no conventional use of a speaker’s body to project an egocentric viewpoint providing “left” and “right” vectors on the basis of which one can say things like “the tree is left of the house,” although there are some uses of projective “front”/“back” terms (e.g., “the tree is in front of/at the back of the house” (from the speaker’s viewpoint; Brown and Levinson, 1992; Levinson and Brown, 1994)^1^. The linguistic emphasis on an absolute frame of reference for spatial description might lead one to expect temporal metaphors based on geocentric coordinates. In Tzeltal expressions for time, there is indeed a spatial metaphor in terms of time extending uphillwards into the future [e.g., “I’ll see you next year,” *ta yajk’ol ach’ ja’wil* (“at its-uphill of New Year,” i.e., just after New Year’s Day)]. However, the relationship between temporal and spatial description is highly variable: there are at least four other distinct schemata of time conceptualization underlying Tzeltal language use: (i) deictic ego-centered time (e.g., with directionals, demonstratives, and locative adverbs), (ii) time as an ordered linear sequence (e.g., “first”/“at the front/top” vs. “later”/“at the back/behind”), (iii) cyclic time (times of the day, yearly cycles, agricultural cycles), and (iv) time as spatial extension (e.g., “lengthened (days)”), spatial location or change-of-state (e.g., “entering/exiting a time period”).

The question I address in this study is this: to what extent do the preferred spatial frames of reference in a particular language and culture – that of the Tzeltal Maya of Tenejapa, southern Mexico – influence the construal of time, as evidenced in linguistic metaphors and in non-linguistic conceptual tasks? In what follows I first sketch the ethnographic context for this study, and then describe the language of space and of time in Tzeltal. In the following section I consider spatial representations of time and space as evidenced in cultural artifacts and events and in gesture. In the final section, I report performance on two structured tasks probing the frame of reference bases for linearizing sequences of events, and consider the implications of the findings for our understanding of the relationship of time and space representations in different linguistic and cultural settings.

### The language and its speakers

Tzeltal is spoken in southeastern Mexico by over 300,000 Mayan speakers. The research reported here was conducted in Tenejapa, a remote community in the highlands of Chiapas, home to some 30,000 Mayans who are primarily subsistence corn farmers. The community is bordered on two sides by communities of speakers of the related Mayan language Tzotzil, and many Tenejapans are partially bilingual in Tzotzil, in Spanish, or in both. The community is undergoing rapid social change, but uses of literacy and of Spanish, though increasing, are still fairly restricted, and Tzeltal remains the language of the home and local village arenas.

The language is mildly polysynthetic, head-marking, with obligatory aspect marking and ergative/absolutive crossreferencing on verbs; ergative also marks possessors on nouns. Spatial language in Tzeltal has been extensively described (e.g., Brown and Levinson, 1993; Brown, 1994, 2006; Levinson, 1994, 2003). Temporal expressions are much less well described, although time has been a major theme in Mayan ethnography (e.g., Leon-Portilla, 1973; Gossen, 1974; Tedlock, 1982).

## Speaking about Space and Time in Tzeltal

### The linguistics of space

Spatial language in Tzeltal utilizes primarily two frames of reference for establishing angles on the horizontal (Levinson, 2003): an absolute frame of reference utilizing the overall slope of the land downhillwards toward the north to project an “uphill/downhill” axis and an orthogonal “crossways” axis on the basis of which objects at all scales are located (e.g., “the machete is standing downhillwards of the doorway”), and (2) an intrinsic frame of reference utilizing body part terms to project an axis, used to describe nearly contiguous spatial relations (e.g., “the man is standing at the car’s front”). There is no systematic use of a relative “left”/“right” system based on coordinates projected from ego’s point of view, although deictic terms (e.g., demonstratives, deictic adverbs, and motion verbs like “come”/“go,” “arrive.here”/“arrive.there”) utilize an egocentric viewpoint. Adult speakers remember and reason about spatial layouts in terms of their absolute coordinates, and they routinely and accurately point in absolute (geographically accurate) directions to identify referents (Brown and Levinson, 1992, 1993, 2009; Levinson and Brown, 1994; Levinson, 2003). Other spatial notions which are less obviously applicable to time are richly lexicalized, including a large set of “dispositional” predicates characterizing spatial properties (shape, size, orientation, distribution) of objects and their configurations (Bohnemeyer and Brown, 2007).^2^

### The linguistics of time

Temporal reference in Tzeltal is coded both grammatically and lexically, with rampant use of spatial words including motion verbs, body part terms, and dimensional terms. Aspect (completive, incompletive, stative), but not tense, is obligatorily marked on verbs; this means that utterances must be anchored in relation to a temporal-aspectual perspective (completed events vs. ongoing events vs. stative events) but not deictically to the time of utterance. In addition, various derivational processes can mark the action of verbs as duratively in progress (inchoative), iterative, etc. Two aspectual particles are frequent in time expressions; like the verb aspect markers these are not applicable to space. The first is *to* “yet, still, until,” the normal way to express future (1) as well as a pre- or post-limit to an event or state change (2)^3^:

(1) ya   to       j-pas      ta        xemona   ya   x-tal-0
  ICP yet/still 1E-do PREP week       ICP ASP-come-3A
  “I’ll do it in the week that’s coming [i.e., future, next week].”
(2) jajch-el-on      to
  arise-NOM-1A yet/still
  “I have just gotten up.” [i.e., “I have just achieved the state of having risen.”]

The second is *ix* “already,” which marks a perspective on an event as having been completed or a change-of-state as having been achieved:

(3) ochotik=ix         ta    agosto ini
  enter-1PLI=ACS PREP August this
  “We have entered August now.”
(4) jelaw=ix     y-ora-il         k’epelaltik
  cross-0=ACS 3E-time-NOM   dry.season
  “The dry season has already passed.”

Two aspectual verbs, *lijk* “begin” and *laj* “finish, die” can specify both spatial (5) and temporal (6) incipience/termination:

(5) ya   x-lijk-0        te    ch’ajan     tak’in    li’i,      ya
  ICP ASP-begin-3A DET cord     metal    here,   ICP
  *x-laj-0          li’i*.
  ASP-finish-3A    here
  “The wire (spatial extent from A to B) begins here (at A), it finishes here (at B).”
(6) *ya   x-lijk-0      ja’al.   ya   x-laj-0=ix*.
  ICP ASP-begin-3A rain.   ICP ASP-finish-3A=ACS
  “The rain begins. It’s (now) finished.”

Similarly, *jil* “remain.behind” applies to both time and space [e.g., *jil ta sna* “he remained behind (spatially and temporally) at his house,” or “the days behind us *jil* “remain.behind”]. In contrast, the word *jal* denotes a long extent of time but not of space: *jal to sk’aalel* “it’s a long time from now” (lit.: “its days extend long”), or *ya xjalaj* “it lasts long.”

#### Time words

A general word for time, *ora*, borrowed from Spanish, is used in certain time expressions: *bi ora* “when” (lit.: “what time?”) or *jayeb ora* “when” (lit.: “how much time?”), *yorail* “its time/season.” In other expressions the word for “sun” *k’aal* extends to “day,” with spatial imagery: *olil k’aal* “noon” (lit.: “middle sun/day”), *mal k’aal* “afternoon (lit.: “sun spills/falls”), *xch’ixil k’aal* “throughout the whole day” (lit.: “its-long.thin.thing day”).

The word *k’alal* is used as a relative pronoun in temporal clauses expressing co-occurring time periods (as in 7) and also spatial extents (as in 8):

(7) 0    *lijk-0      ta      sab,     te       k’alal   a*
  CMP begin-3A PREP morning COMP when   CMP
  *sak-ub-0         tal*.
  white-INCH-3A DIRcome
  “They left in the morning, when it was dawning.”
(8) ben-0      bel        k’alal   jobel
  walk-3A DIRgo when   PLACE
  “He walked all the way to San Cristobal.”

In Tzeltal, as in Yucatec Maya (Bohnemeyer, 2002; Le Guen, under review), there are no words translatable as “before” and “after.” The nearest equivalents are constructed from the spatial body part words *ba* “forehead/top” and *pat* “back,” from which come *babi* “first (in a spatial or temporal sequence)”^4^ and *ta patil* “at (its) back, i.e., later,” respectively:

(9) babi   ya   x-ba    k-il     wakax,    patil   ya    x-tal-on
  first   ICP ASP-go 1E-see bull    later   ICP     ASP-come-1A
  ta     a’tel      li’i
  PREP   work   here
  “First I’ll go see my bull, later I’ll come to work here.”

With this repertoire of time words, and others, time is conceptualized in different – sometimes overlapping, sometimes opposing – frameworks in Tzeltal.

#### Deictically anchored time vector

The directionals *tal* “coming (toward speaker)” and *bel* “going (awaywards)” are used to express spatial movements or static arrays oriented toward speaker or away from speaker (or other deictic center) with no directional vector other than that of time toward/away from speaker (or deictic center). These are employed also in temporal expressions, as in (1) and (7) above, and in (10) and (11):

(10) la      j-pas-tik=ix     ja’ i        xemona    0     k’ax-0
  CMP 1E-do-1PLI=PT    !   DEI week    CMP    pass-3A
  tal      i
  DIRcome DEI
  “We finished doing it (during) this week that’s passed by coming.” (i.e., the week just before the one we are in now, reckoning from the past toward us in the direction of now).
(11) s-k’an    to   bel         wakeb  u         te      k’epelaltik=e
  3E-want still DIRaway seven   month   DET dry.season=CLI
  “It’s still six months till the dry season.” (reckoning awaywards from here/now – *bel* – into the future).

**Figure 2:**
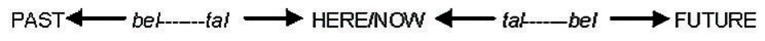
**Time as a vector in relation to deictic center**.

The frame of reference associated with the deictic *tal*/*bel* terms is a relative one, symmetric in past and future. In (1) the future event expressed with *tal* is construed as approaching “now,” in (10) the past event is in a week whose passing is construed as approaching “now”, and in (11) the future months are construed as awaywards from “now.” This conceptualization can be schematized as in Figure 2, as a vector with time periods in the past construed as approaching from the speaker’s perspective “now” (the reflection type of relative frame of reference) and those in the future construed as receding “awaywards” from “now” (the translation type of relative; Bender et al., 2010).

This construal is on analogy with spatial descriptions which characterize a trajectory in relation to speaker’s current location (e.g., a route direction toward or away from “here”):

(12) ya    x-tal-0         li’i   ta    sab,       ta    patil
  ICP  ASP-come-3A    here  PREP  morning, PREP   later
  ya   x-lok’-otik       bel      ta     jobel
  ICP ASP-exit-1PLI   DIRaway   PREP   PLACE
  “He (will) come here in the morning, later we’ll set off [lit.: “exit awaywards”] toward San Cristobal.”

The deictic demonstrative *ini* “this, here” and adverb *li’* “here” in collocation with time expressions also pick out time periods in relation to current speaker’s time/place of speaking:

(13) ta    ora      ya’tik   ini
  PREP  now/hour today    this
  “right now (i.e., right at this moment)”
(14) *ya   j-pas-tik      li’    ta    j-ajk’/      jun xemona*.
  ICP 1E-do-1PLI here PREP NC-moment/one week
  “We’ll do it “here’ (i.e., precisely) in a moment/in a week” (where “here” is temporal, not spatial, emphasizing closeness to “now”)

#### Time as a deictically anchored static sequence of time periods

**Figure 3:**
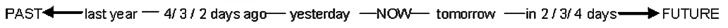
**Time periods lying on a vector awaywards from deictic center**.

Although there is no grammaticized tense in Tzeltal, with adverbs one can discriminate a sequence of deictically anchored periods on a highly differentiated one-dimensional time line. From the time point of *ta ora ya’tik ini* “right now,” one speaks of time extending into the past with adverbial expressions: *ajk’ nax* “a moment ago’, *sab nax* “just (this) morning,” *woje* “yesterday,” *cha’je* “two days ago,” *oxeje* “three days ago,” *chaneje* “four days ago,” *junabe* “a year ago,” *namej* “long ago (many years).” Symmetrically, one speaks of time extending into the future from *ya’tik* “today (now)” with adverbs like *ta ajk’/ta tz’in* “in a moment,” *pajel* “tomorrow,” *cha’we* “day after tomorrow,” *oxej* “three days from now,” *chonej* “four days from now,” *li’ to ta waxakeb k’aal* “here in eight days,” etc. This construal is like the first deictically anchored one except that it lacks any motion; the frame of reference is relative, extending symmetrically awaywards from the deictic origo as diagrammed in Figure 3.

#### Cyclic time

**Figure 4:**
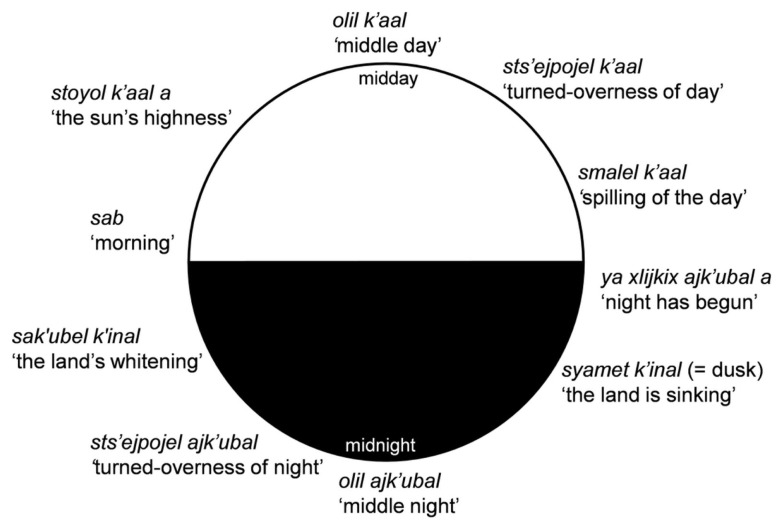
**Diurnal time in Tzeltal**.

Time conceptualized as a cyclic sequence is encoded in sets of words for the diurnal cycle, the months of the year (either the 20-month traditional Mayan calendar or the 12-month modern calendar), and the seasons. Diurnal cycle terms use the words *k’aal* “sun, day,” *ajk’ubal* “night,” *sab* “morning,” and *k’inal* “land” in nominal or verbal expressions to capture the different culturally relevant time periods in the diurnal cycle (see Figure 4). Within living memory of everyone over about age 30, watches and clocks were rare, and rising in the middle of the night for meetings or to catch a bus to town were events gauged by these divisions of the night and day, by the position of the sun or the stars.

Cyclic time construal is also evidenced in how Tzeltal speakers talk about the change-over of years in terms of the change-over of religious offices: *jelonel* “exchange, turn-over” is the metaphor for the New Year, and for the replacement of last year’s incumbents for the new set of cargo holders, as well as the replacement of a prior generation by a new one [*yakal ta jelonel* “they are in the process of replacing (them)”]. Generations are construed as cyclic in the sense that grandfathers are reinstantiated in grandsons; first grandsons traditionally receive the name of their paternal grandfather. Another metaphor – non-cyclic – for generations is sequential layers piling up or exchanging themselves: *slamal-lam* “layerings”: *jlam jmamtik* “one layer (for) grandparents,” *jlam jme’tat* “one layer (for) parents,” *jlam jo’otik* “one layer (for) us,” *jlam jnich’nab* “one layer (for) our offspring,” *yu’un jelel* “because of exchanging (themselves).” Lower layers are earlier in time.

A cyclic view of time and space is implicit in traditional tales of mythological journeys, for example the travels of Tenejapa’s founding saint Kajkanantik around the boundaries of the community – a circumnavigation reproduced in cyclical ritual journeys to the sacred mountains.

#### Time as change-of-state or location along a unidirectional time line

A different set of metaphors represents time in terms of an unoriented sequence, for example with the body part metaphors *ta sba* “first” (lit.: “at its top/forehead”) vs. *ta patil* “later” (lit.: “at its back”), as in (9) above, indicating placement in a sequence without reference to a deictic origo or any spatial directionality. This is equivalent to Moore’s (2011) metaphor “sequence is relative position on a path,” and to Bender et al.’s (2010) intrinsic frame of reference.

The same construal appears in metaphors where time periods (e.g., years, ages, school classes, religious offices) are expressed as locations or as the result of change of location or state, for example in terms of containers sequenced along a time line; one “exits” from one earlier in the sequence and “enters” one later:

(15) lok’=ix    ta         cheb  ja’wil,   och=ix    ta        oxeb    te
  exit=ACS PREP  two   year    enter=ACS PREP three DET
  alal=e
  child=CLI
  “The child has exited two years (of age), he has entered three.”
(16) ja’ tik’    waxakeb    k’aal   li’     ta      martextik   ya
  !   insert   eight    day     here    PREP   Wednesday    ICP
  x-tal-0      i
  ASP-come DEI
  “It is a week (from today) on Tuesday (when) he’ll come.”
  [*tik’* as a verb means “insert.into.container”; the image evoked is of a container full with a week (“eight days”) by the time he comes].

As in many languages, “long/short” and “near”/“far” spatial terms can also apply metaphorically to time points and periods: *najt xkuxlejale* “his (a child’s) growing-up (time) is long,” *tijilix yoral* “its time (is) near,” *nopol olil k’aal* “near midday,” i.e., about 11 a.m.). These can be taken as demarking the beginning/ending of time periods construed as containers:

(17) nopol   s-k’an    x-lok’-0     jo’winik ja’wil   te      0
  near    3E-want ASP-exit-3A fifty    year   COMP CMP
  jelaw-0   k’op     li’=e
  pass-3A    fighting   here=CLI
  “It’s nearly 50 years ago (lit: “it wants to exit near 50 years”) that the fighting passed by here.”

#### Time as a unidirectional vector oriented “uphillwards”

The future as upwards or uphillwards is a change-of-state or location metaphor, using an absolute frame of reference (Bender et al., 2010), with the time line in both past and future established as an oriented “down”/north) → “up”/south/vector metaphorically anchored in geographical space. This is a field-based metaphor in Moore’s (2011) terms. These metaphors draw on the Tzeltal vocabulary dedicated to the spatial absolute system, consisting of verbs (“ascend”/“descend”/“go.across”), directional adverbs (“ascending”/“descending”/“going.across”), and nouns (“uphill”/“downhill”/“acrossways” and “at.its.underneath”/“above.it”). For example:

(18) *tame    ta    j-pat-tik       ya   j-kajtaj-tik*,
  if    PREP 1E-back-1PLI   ICP 1E-count-1PLI,
  koel       ya   j-kajtaj.    koel       bel       a       ta’yej
  DIRdown ICP 1E-count DIRdown DIRaway    ANA PT
  “If backward (into the past, lit.: “to our backs”) we count, downwards I count. Downwards awaywards in that case.”
(19) *ja’  y-anil                 abril    te      marzo=e*,
  !   3E-underneath/downhillwards April DET March=CLI,
  ja’  y-ajk’ol            abril
  !   3E-above/uphillwards    April
  “[In the sequence of months] March is downwards of April, April is upwards.”
(20) alan    ya   s-k’an    ya   s-na’    s-toj-ol
  downhill ICP 3E-want ICP 3E-know 3E-straight-NOM
  “Downhill [i.e., ahead of the event] he wants to know.”
(21) alan    k’ub-an-bil              we’el-il
  downhill ask.ahead-TVR-PASSPT food-NOM
  “The meal was prepared “downhill” (ahead of time).”
(22) *moel         ya   x-ben-0       y-u-il*,
  DIRascend ICP ASP-walk-3A 3E-month-NOM,
  ya   x-mo-0         bel         te      ja’wil=e
  ICP ASP-ascend-3A DIRaway DET year=CLI
  “The months go upwards, the years ascend awaywards.”
(23) s-kaj-al-kaj             ya   x-tal       jujun    u
  3E-be.mounted.on-Vl-REDUP ICP ASP-come each   month
  “Layer by layer each month comes.” [i.e., with the positional root *kaj* “be.mounted. on” an upwards direction is introduced to the construal of how months succeed one another as layers]
(24) ya   j-mo-tes-be-tik/          ko-tes-be-0
  ICP 1E-ascend-CAUS-DIT-1PLI/    1E-descend-CAUS-
         s-k’al-elal    te      junta=e
  DIT-3A     3E-day-NOM DET meeting=CLI
  “I raise/lower the date for the meeting.” [i.e., make it later/earlier]
(25) ya   x-sujt-on         bel        ta    y-anil       k’in
  ICP ASP-return-1A DIRgo PREP 3E-underneath   fiesta
  *santziako*.
  Santiago
  “I’ll return just before [lit.: “below”] the fiesta of Santiago.”
(26) *moel        ya   j-bil-tes           j-nich’nab:    Alux* (oldest),
  DIRascend ICP 1E-name-CAUS 1E-offspring: Alux
  *Manel, Petul, Xun, Mikel, Marta* (youngest)
  Manel  Petul  Xun  Mikel  Marta
  “Uphillwards I name my children: Alux, Manel, etc. (named in order of their birth events, not in descending order of their ages, which would be *koel* “downwards.” Lowest is oldest, and successive child-arrivals are construed as ascending).^5^

In most of these metaphors the uphill/downhill axis is the salient one, in some, however, it is the vertical axis. Co-occurring gestures may disambiguate the axis. Furthermore, for some contexts this “down-up” metaphor is asymmetric; for example some speakers accept sentence (25) as meaning “before the San Tziako fiesta” but are unwilling to accept the “after” version *ya xsujton bel ta yajk’ol k’in* “I’ll return above/after the fiesta” or *ta spat k’in* “at the fiesta’s back,” preferring *ya xsujton bel ta slajel k’in* “I’ll return after [lit.: “at the end/finish of”] the fiesta.” Thus not all down-up time metaphors are equally idiomatic in this community.

Note that the direction of the time vector in Tzeltal – with future uphill – contrasts with that reported for Mandarin Chinese, which also uses an “up”/“down” metaphor for time but with the vector pointing downwards into the future (Traugott, 1975).

To summarize: linguistic metaphors for time draw on spatial language in the two frames of reference used in Tenejapa, the intrinsic system of body parts (especially “front/back”) and the absolute system of “uphill/downhill’ terms. They also employ deictics and directionals for expressing time in relation to the here and now, “long”/“short” terms for temporal extents, and “near”/“far” terms for the distance of one event from another. The majority of temporal expressions except time period words (e.g., “hour,” “year,” weekday, month, and fiesta names), aspect markers and some verb semantics draw on space, and no source domains other than space are apparent in the over 150 time expressions I elicited [for example, there were no metaphors like the English “time is money” or Aymara “knowledge is vision” (Núñez and Sweetser, 2006)].

### Spatial representations of time

Cultural knowledge structures and practices of various kinds provide indirect evidence for how space is mentally represented and extended to the temporal domain in this community. Here I discuss three.

#### Cultural artifacts and events

The ancient Maya had sophisticated calendars and elaborate ways of reckoning time in cycles; their modern descendents still use remnants of these to varying degrees and can if pressed represent them diagramatically (Gossen, 1974; Vogt, 1976; Tedlock, 1982). Many Tenejapans over the age of 40 or so still use the 20 ancient Mayan calendar months for calculating planting times and rituals, although for the younger schooled generations these have been largely replaced by watches, clocks, and modern calendars. Both ancient and modern systems utilize numbers, allowing time to be quantified in discrete chunks. But aside from setting planting schedules and establishing the yearly cycle of ritual events, in everyday non-specialist contexts this time-counting ability did not, and still does not, find much cultural use. Until recently Tenejapans paid no attention to their dates of birth (used only for interactions with Mexican authorities), and they reckoned past times in terms of memorable co-occurring events, for example pinpointing when the great locust plague came (in the 1950s) by how big a child one was at the time. Tenejapans traditionally reckoned times for past, current, and future events by the sun’s position and by the size and placement of their shadows. Time reckoning in terms of events, rather than “Time as Such” (Sinha et al., 2011) seems to be the cultural preference.^6^

#### Writing systems

Within the past 20 years school attendance has dramatically increased, with most Tenejapans now completing at least the sixth grade. Some go on to high school, but for most, education stops there and regular use of literacy and Spanish is only for those who leave the local community for work in the surrounding Mexican towns. Literacy is only in Spanish (with a handful of anthropologist-trained exceptions); books – except for the Bible – are largely absent from homes, and uses for reading or writing in the local villages are minimal. Only one of the participants in our time/space tasks was functionally literate.

#### Gesture

As absolute speakers in the spatial domain, Tenejapans’ spatial gestures are geocentrically anchored – they point regularly in geographically accurate directions to indicate referents even for far-distant places and events (Levinson, 2003). Geographically accurate pointing and the correspondingly necessary impressive dead-reckoning skills are well documented for several other Mayan groups, even in the absence of co-occurring language using an absolute frame of reference (Haviland, 2003, 2005 for the Tzotzil Maya; Le Guen, 2011a,b, under review, for the Yukatek Maya). Brown and Levinson (2009) argue that the reliable geographic accuracy of gestures accompanying spatial language is an important factor in Tzeltal children’s early acquisition of the absolute spatial language system.

Gesturing for temporal reference is more limited, but people routinely point to locations in the sky to indicate the time of day being discussed by where the sun would be at that time. I have also occasionally observed metaphorical pointing, with Tenejapan individuals pointing backward over their head or shoulder to indicate past times. This contrasts with Le Guen’s (2011a,b, under review) claim, based on his Yukatek Maya observations, that users of an absolute system for gesturing cannot exploit gestural space when expressing time, as the concrete spatial interpretation – of a gesture to something in “real” geographic space – in all directions around the body preempts any temporal interpretation. Tenejapans are able to tolerate this ambiguity, at least in some contexts. This issue of the relationship between predominant frames of spatial reference and metaphorical directionality in gesture needs further systematic investigation in both communities.

## Tzeltal Representations of Time in Field Tasks

Is the plethora of space-to-time mappings in the language reflected in conceptual preferences when Tzeltal speakers are thinking about time non-linguistically? This question was explored in two L&C Field Manual tasks (Boroditsky et al., 2008) in which consultants were asked to map temporal sequences onto spatial locations in such a way as to reflect the temporal progression portrayed. The linguistic metaphors for time in Tzeltal which prominantly include a “time progesses uphillwards” conceptualization, along with the cultural practices around time reckoning in this society, suggested the following hypothesis to be tested:

An absolute frame of reference will predominate in Tzeltal spatializations of temporal sequences.

### Materials and methods

Twelve subjects, 6 male, 6 female, with an average age of 52 years (range 39–65) participated in both tasks. The highest level of education of subjects was sixth grade. All but two were multilingual to some degree, with nine speaking some Tzotzil, seven speaking some Spanish. One was literate in Tzeltal, three others said they can write it “a bit.” The tasks were run, in Tzeltal, outdoors on the patio space in front of each participant’s house. None of the participants had any experience with this type of task.

#### Task 1: card arranging

Eight sets of round laminated cards, each set composed of four photos depicting stages in a life cycle (e.g., an egg, a chick hatching, a baby chick, and a grown chicken) or an event developing through time (e.g., a woman at successive stages of pregnancy, or four stages of a banana being eaten) were given in randomized order to subjects who were asked to set them down “showing the order of what is portrayed in the pictures from what happened first to what happened later.” The experimenter was careful to share the same perspective (face in the same direction) as the subjects and to avoid gesturing or using any spatial language that might influence responses. Subjects were free to array the cards in any configuration and direction they chose. In order to disambiguate absolute (up/down) responses from relative (left/right) ones, the task was interrupted after four of the sets had been ordered and the facing direction of the subject was rotated 180° for the final four sets. All sessions were videotaped, the arrays subjects produced were photographed, and subjects’ facing direction and the axial (compass) direction of the resultant temporal sequences were recorded.

#### Task 2: abstract time-point ordering

This task was designed to test the spatialization of abstract time relations, and followed immediately after Task 1. Task materials comprising 14 sets, each set composed of three Tzeltal words or expressions denoting different points in a temporal sequence (e.g., “yesterday,” “now,” “tomorrow”) were constructed, grouped into two groups of seven sets each (see Table 1). A pilot study had revealed that subjects in this population could not interpret instructions to point abstractly to locate time periods in space; the original Field Manual task was therefore adapted using concrete physical objects to represent abstract times (Boroditsky et al., 2008). The experimenter set down a blank round card on the ground directly in front of the seated subject, saying the Tzeltal equivalent of, e.g.: “If I tell you that “today” is here (where I’ve put the card), where would you place “yesterday?” (handing the subject a second blank card) and “Where would you place “tomorrow?” (handing a third blank card). The subject placed these two cards relative to the pre-given mid-time-point card, again with the experimenter sharing the subject’s perspective and with no constraints as to direction or configuration of placement. The order of presentation of the triplets was randomized; after presentation of the first set of seven, the subject was rotated 180° and the second group of seven triplets was presented. All sessions were videotaped, the arrangement produced in each trial was photographed, and compass points were registered for each group of sets. Finally, subjects were asked to point in the “left”/right,” “uphill”/“downhill”/“across,” and “sunset”/“sunrise” directions, to check the accuracy of their understanding of these spatial terms.

**Table 1:** **Abstract time period triplets in Task 2**.

Earliest	Midpoint	Latest
**SITTING 1**		
*woje* “yesterday”	*ya’tik* “today”	*pajel* “tomorrow”
*namej* “long ago”	*yorail ya’tik ini* “nowadays’	*li’ bel pajel cha’weje* “2–3 days in the future”)
*te xemona k’axix a* “last week”	*xemona ini* “this week”	*li’ to ta yan xemona* *bel* “next week”
*yorail ja’lel k’inal ta yan ja’wil* “previous year’s (wet) season”	*yorail k’epelaltik* *ini* “this dry season”	*yorail ja’leltik bel* “next (wet) season”
*sab* “morning”	*olil k’aal* “midday”	*mal k’aal* “evening”
*te yorail k’alal ya xbajt ta wayel* “when you are going to bed”	*te yorail k’alal ya xwayat* “when you are sleeping”	*te yorail k’alal ya xjajchat* “when you wake up”
*tajimal k’in* “Carnival fiesta” (in February)	*k’in santziako* “fiesta of Santiago” (July, current month of study)	*jalame’tik* “Holy mother’s fiesta” (in September)
**SITTING 2, ROTATED 180°**		
*martextik* “Tuesday”	*merkolextik* “Wednesday”	*jwevextik* “Thursday”
*te k’alal alalat to* “when you were a baby”	*a’wa’wilal ya’tik ini* “the age you are now”	*te bi ora mamalatix/me’elatix a* “when you will be an old man/old woman”
*te yan u k’axix a* “last month (April)”	*yuil ini* “this month (May)”	*yuil ya to xtal* “next month (June)”
*junabe’* “last year”	*ja’wil ini* “this year”	*li’ to ta yan ja’wil te ya to xtal* “next year”
*lok’ib k’aal* “sunrise”	*olil k’aal* “noon”	*malib k’aal* “sunset”
*yamal k’inal* “dusk”	*olil ajk’ubal* “middle of the night”	*sakub k’inal* “dawn”
*jajch* “get up”	*pas waj* “make tortillas”	*we’ waj* “eat”

### Results

#### Task 1: card arranging

There are 16 possible coherent strategies for sequencing, depending on (1) whether the frame of reference for establishing a direction for the sequence was geographically based (absolute) or viewpoint based (relative), as indicated by whether the direction of the array changed when facing direction changed, and (2) the basis for the direction used (east/west or north/south for absolute, left/right or direction in front, and near-to-ego/farther-from-ego for relative). The results are shown in column 2 of Table 2, which gives the number of responses manifesting the different strategies for each subject (labeled s1, s2, etc.). The table reveals a high level of between-participant variation and a lower but substantial within participant variation. Five of the 12 subjects were 100% consistent in their own responses across trials in this task, but they used five different strategies for representing the time vector: three relative strategies (one left to right, one right to left, one near-to-far in front) and two absolute ones (one south to north, one east to west). Of the others, two were so inconsistent as to be uncodable. The other five shifted their strategies across turns: the predominant responses were two left to right, one right to left, one far to near, and one ambiguous between relative left to right and absolute west to east.

**Table 2:** The predominant strategies of subjects (s1–s12)*.

Ordering strategy	Task 1 (8 trials)	Task 2 (14 trials)
**ABSOLUTE**		
Uphillwards (south to north)	s1 (100%)	–
Sunrise to sunset (east to west)	s12 (100%)	s9 (50%)
West to east	–	s6 (79%)
Vertical down to up	–	s7 (100%)
**RELATIVE**		
Left to right	s8 (100%), s7 (75%), s5 (50%)	s8 (100%), s11 (71%), s1 (64%), s5 (50%)
Right to left	s9 (100%), s11 (75%)	s4 (100%)
Near to far	s3 (100%)	s3 (79%)
Far to near	s10 (50%)	–
Midpoint far left, past middle, future far right	–	s2 (79%)
Uncodable	s2, s4, s6	s10, s12

*Predominant = used in at least 1/2 the trials and in at least 1 more trial than any alternative strategy. % are for aggregated numbers across all trials for each task.

In short, in this task there was no consistent basis across subjects for mapping temporal sequence onto a spatial frame of reference.

#### Task 2: abstract time-point ordering

Again, a wide variety of strategies were in evidence, and subjects did not necessarily use the same strategy as they had used in Task 1. Two new directional strategies appeared in this task: the time vector represented as (1) a vertical stack (with past on the bottom, future on top) and (2) west to east, counter to the sun’s path. The results for each subject (s1–s12) are summarized in column 3 of Table 2.

Given the large amount of variation, we cannot provide any statistical assessment of these results. Yet it is clear that in both tasks, consultants felt free to construe the directionality of these temporal sequences in terms of vectors based in differing frames of reference. Except for two consultants (s5 and s8), there is a notable absence of any consistent tendency to use left-to-right ordering, reflecting the minimal literacy levels of this group. This contrasts strongly with the consistent left-to-right performance of English speakers and the consistent right-to-left pattern displayed by Hebrew speakers on this kind of task, consonant with the direction of their writing systems (Bergen and Chan Lau, 2012). For only two subjects is there a clear directional preference displayed across both tasks: for s8 for a left-to-right solution, for s3, near to far; both subjects are female, and both were minimally literate, although they had completed 5 or 6 years of schooling. The variability in the Tzeltal results is comparable to findings for tasks of this kind in some other studies (see Torralbo et al., 2006 for English; Fuhrman et al., 2011 for Mandarin; Bender et al., 2010 for Tongan; Le Guen, under review for Yukatek Maya).

A clearer picture can be obtained if we set aside the data where subjects’ responses display either no coherent strategy (the uncodable cases) or strategies that are incompatible with any licensed by linguistic form and practice (i.e., the cases of west to east, right to left, far to near, and zigzag from middle to left to right). We can then examine the raw data for just those cases where subjects’ performance on these tasks display a predominant strategy compatible with the language data, namely absolute (oriented by a vector extrinsic to the task situation) and relative (ego-perspective based). Table 3 collapses the raw data (pooling subjects who responded the same way) into the two types of frame of reference predicted by the language usage to be available in this community: absolute (ABS; the data for the five subjects who used absolute strategies, namely, s1 on Task 1, s6, s7, s9, and s12), and relative (REL; the data for the five who used relative (REL) strategies: s1 on Task 2, s5, s7, s8, and s11). Table 3 shows that our hypothesis of a preference for using absolute strategies in these tasks is clearly disconfirmed. Indeed, the reverse is the case, although given the small numbers and small proportion of the total data set, this result is only suggestive.

**Table 3:** **Coherent ABS and REL responses compared**.

	Task 1	Task 2	Crosstask Totals
ABS uphillwards (N → S)	(8)	(0)	(8)
ABS sunrise-sunset (E → W)	(8)	(7)	(15)
ABS vertical (down → up)	(0)	(14)	(14)
Total ABS	38% (16)	29% (21)	33% (37)
REL left → right	(18)	(40)	(58)
REL near → far	(8)	(11)	(19)
Total REL	62% (26)	71% (51)	68% (77)
Total ABS + REL	(42)	(72)	(114)

*% = proportion of total responses across the subset of data where responses display an absolute or relative strategy (the top half of Table 2). For Task 1 n = 5 (the data of s1, s5, s7, s8, s12); for Task 2 n = 7 (the data for s1, s5, s6, s7, s8, s9, s11).

It is clear from these results that the prolific use of absolute “up/down” linguistic metaphors in Tzeltal time expressions is not reflected in most subjects’ responses on these time spatialization tasks. Yet there were some hints at absolute thinking: most subjects changed sequence alignment on the second sitting, and many angled the sequence to align better with a N/S or E/W angle. Only one subject, in contrast, was consistently left to right in her responses on both tasks.

## Discussion

So, is the future “up” or “uphill” in Tzeltal? Yes and no. “Yes” in the sense that many linguistic expressions rely on this metaphor; a dominant frame of reference for describing spatial relationships in this community is indeed sometimes employed in the metaphorical description of time. But “no” in the sense that (1) time progressing “uphill” is not the only, nor even the predominant metaphor (in terms of usage frequency) in linguistic time expressions, and (2) in the time-sequence ordering tasks, speakers used a variety of directional bases for the vectors motivating their time orderings, with most individuals displaying remarkable inconsistency across trials. Assuming (and this is by no means sure) that performance on these tasks reflects, at least some of the time, a spatial frame-of-reference basis for selecting a time direction, it would seem that in this data there is no clear correlation between metaphorical mappings between space and time in linguistic representations and those reflected in the cognitive perspectives adopted in these tasks. Certainly, the multiplicity of schemata for time expression in the Tzeltal language affords a range of possible construals, yet in the two non-linguistic tasks the vectors utilized to convey earlier-to-later time points include some directions not exploited at all in the linguistic system, for example, time vectors pointing downhillwards, or from west to east, or from right to left. Nor do there seem to be any aspects of the cultural or linguistic context which could readily explain using such linguistically unlicensed vectors for representing temporal sequence. This data suggests the likelihood that the structure of the tasks – requiring subjects to spatialize time sequences by spreading them out in space – was not entirely natural for all the participants.

The results are in a sense the opposite of that found in another predominantly absolute language, the Australian Aboriginal language Kuuk Thaayorre (Boroditsky and Gaby, 2010; see also Gaby, under review). In that context, the results of the same two experimental tasks showed Kuuk Thaayorre speakers to consistently represent time as flowing from East to West, as their spatial linguistic repertoire would lead one to predict. Yet this absolute space-time mapping was restricted to non-linguistic cognition and co-speech gesture; their oral descriptions of time did not use absolute directional terms at all. In the Tzeltal case, in contrast, the multiplicity of schemata for construing time linguistically is parallel to, but does not exhaust, the multiplicity of schemata for sequentially arraying temporal progression in non-linguistic tasks. Time thus appears to be more open to alternative perspectival construals than space is in this community. This suggests that, although languages vary widely in the set of spatial terms and reference frames habitually used to talk about space, those that are available – or even preferred in spatial description in the language – do not rigidly determine the frames of reference used for time.

This study provides clear evidence for a further spatial metaphor – “time moves uphillwards” – to add to the burgeoning literature on crosslinguistic variation of time construals. But it provides no support for the hypothesis that this metaphor has an effect on non-linguistic cognition. Future work should pursue an explanation for these two findings: (i) the unexpected apparent dominance of relative strategies in the non-linguistic tasks and (ii) the extreme crossindividual variability in performance. In particular, the puzzle of why some participants systematically use a particular spatial frame-of-reference basis for selecting a time direction and others apparently do not, needs to be investigated. Our interim conclusion must be that, despite the usefulness of spatial concepts for thinking about the more abstract domain of time, there is no automatic transfer of spatial frames of reference to those for time.

## Conflict of Interest Statement

The author declares that the research was conducted in the absence of any commercial or financial relationships that could be construed as a potential conflict of interest.

## Footnotes

^1^This is was still true in 2008 in the community of Majosik where my work has taken place. The pattern is, however, changing under the pressure of modernization and increasing Spanish usage. Polian and Bohnemeyer (2011) report results of spatial tasks in neighboring hamlets, and in the town center, showing evidence that some projective “left”/“right,” and a relative frame of reference, are used.

^2^Only one of these (*kaj* “be mounted on”) appears in my database of time expressions. There are also spatially rich verbs for characterizing motion, including “affective verbs” which portray spatial and motion gestalts.

^3^I use a practical orthography for Tzeltal, where j = [h] and indicates a glottal stop or glottalized consonant. The following abbreviations are used in interlinear glosses: 1/2/3 – first/second/third person; 0 – null morpheme; E – ergative, possessor; A – absolutive; ACS – achieved change-of-state clitic; ANA – anaphoric particle; ASP – neutral aspect; CAUS – causative; CLI – clause-final clitic; CMP – completive aspect; COMP – complementizer; DEI – deictic particle; DET – definite determiner; DIR – directional; DIT – ditransitive; EXIST – existential predicate; ICP – incompletive aspect; INCH – inchoative; NC – numeral classifier; NOM – nominalizing suffix; 1PLI – 1st person inclusive plural; 1PLE – 1st person exclusive plural; PL – 2nd/3rd person plural; PREP – generic preposition; PLACE – place name; PT – discourse/evidential particle; TVR – transitivizing suffix; ! – proposition affirmation (“it is the case that”).

^4^The partially equivalent word *nail* “first” means only temporally first; it is not applicable to spatial precedence.

^5^In the same way, reading out a list of names (e.g., summoning men to communal work) is done “*moel*,” upwards into the future (*moel ya xlok’ sbil ta lista* “ascending their names exit from the list”), rather than “*koel*,” vertically downwards as they are listed sequentially on the paper from which they are read. Future *moel* overrides spatial *koel* in this case.

^6^Sinha et al., 2011, p. 163) argue that in Amondawa, an Amazonian language and culture, “time reckoning is apparently entirely absent from the repertoire of cultural practices.” They account for this in terms of the absence of a number system or any other linguistic tool that could be used to count chunks of time; instead, the Amondawa reckon time in relation to events.
